# An Exploration of the Possibilities of Current Mental Health Services to Tackle Transgenerational Effects of Parental Mental Illnesses on Offspring Adjustment in Lithuania

**DOI:** 10.3389/fpsyt.2021.764394

**Published:** 2021-10-29

**Authors:** Sigita Lesinskiene

**Affiliations:** Clinic of Psychiatry, Institute of Clinical Medicine, Faculty of Medicine, Vilnius University, Vilnius, Lithuania

**Keywords:** children, mental health, mental disorders, parents, services, transgenerational effects

## Abstract

The topic of mental health and mental disorders is very sensitive and delicate in families and the society. Stigma is one of the main reasons for little help-seeking for mental disorders. Transgenerational effects of mental disorders is the utmost sensitive theme that brings difficulties for service organization and research. By emphasizing the importance of the effects of parental mental illnesses on the health of offspring and their adjustment, together with sharing the international experience between professionals, both the administration of services and society could provide opportunities for further positive change in this little-studied but utmost actual field. There is still a lack of appropriate long term systematic programs and ways to overcome complex organizational challenges. Sharing international experience and research could help find ways that best fit the situations in each country. After a descriptive analysis of the current system of mental health services in Lithuania, opportunities were sought to meet the needs of children and adolescents whose parents have mental disorders by ensuring their healthy psychosocial development. Child and adolescent psychiatry services are a more favorable and less stigmatized area in Lithuania than adult psychiatry, so assistance and specialized programs for children of parents with mental health problems could be organized using the country's relatively well-developed network of child and adolescent psychiatric and pediatric services. For such a small country with limited resources, there could be a possibility to use and strengthen the existing network of services together with finding opportunities for mixed models of financing and cooperation with non-governmental initiatives and organizations. A unique network of primary mental health outpatient centers that provide services for adults and children/adolescents could serve as a reasonable basis for the systematic implementation of specialized programs and initiatives in this field. This network is still not adequately used in the organization of mental health prevention, early intervention, and complex treatment services for the children of parents with mental illness.

## Introduction

The actuality of the theme is clearly highlighted in the JM REY'S IACAPAP e-Textbook: “Children of parents with mental illness (COPMI) face multiple psychological, biological and social stressors that increase their personal risk for the development of mental disorders. Still, health systems in many countries do not allow for preventive measures needed for COPMI and their families. Moreover, data exist mostly for high-income countries, whereas we have almost no information about the situation of COPMI in low- and middle-income countries, where the majority of children live” (1).

Rehm and Shiled (2) noted that “mental and addictive disorders affect a significant portion of the global population with a high burden, in particular in high- and upper-middle-income countries. The relative share of these disorders has increased in the past decades, in part due to stigma and lack of treatment.” Meta-analysis and a meta-regression on prevalence changes (3) in adult mental illness since the 1970s “have found evidence of a small but significant increase over time (OR 1.18).”

The Department of Mental Health and Substance Use of the World Health Organization (WHO) encourages countries to implement “a life course approach: policies, plans and services for mental health need to take account of health and social needs at all stages of life, including infancy, childhood, adolescence, adulthood and older age” (4). WHO Mental health action plan 2013–2020 emphasizes, in particular, the importance of early age: “Childhood and adolescence are critical stages of life for mental health and well-being. This is when young people develop skills in self-control, social interaction and learning. Negative experiences – at home due to family conflict or at school due to bullying, for example – have a damaging effect on the development of these core cognitive and emotional skills. The socioeconomic conditions in which children grow up can also have an impact on their choices and opportunities in adolescence and adulthood” (5).

Findings of a register-based nationwide cohort study in Denmark (6) indicate that “offspring of parents with severe mental illness experienced increased mortality and somatic morbidity, warranting heightened vigilance and support for this population.”

Research indicates that parents' mental illnesses negatively impact their children's health (both physical and mental), learning, cognitive performance and behavior, and increases risks of child maltreatment, injury, socioeconomic adversity, attempted suicide, and violent criminal offending (7–13). Studies emphasize the importance of reaching and addressing children whose parents have mental health problems for preventative and treatment purposes (14–17). Approaches and developments in this field vary depending on the country, from a variety of available interventions to still existing shortcomings in the services.

This study aimed to analyze mental health service provision in Lithuania and explore the gaps and developments in preventing and treating mental health problems in the offspring of mentally ill adults.

## Materials and Methods

The situation was described using both qualitative and quantitative research findings together with the author's views and insights and divided into four steps: (1) An introductory overview of the theme and main risks for COPMI; (2) A descriptional analysis of CAP services in Lithuania; (3) An up-to-date narrative review of initiatives and programs that exist in the country to help COPMI; (4) An elaboration of the recommendations for the further developments of existing services in the country in order to have more parents and their offspring reached and aided in future.

## Results

### Child and Adolescent Mental Health Services in Lithuania

In Lithuania, child and adolescent psychiatry (CAP) is a separate medical speciality that focuses on preventing, diagnosing and treating psychopathology in children and adolescents; it covers the provision of mental health services for children and adolescents (CAP services). CAP has a long tradition (more than 60 years) in the country with roots in pediatrics and psychiatry. Two universities in Vilnius and Kaunas offer 4-year fellow programs for CAP after general medical studies. The Lithuanian Society for CAP has over a hundred members. Intersectoral collaboration is an essential yet complex and challenging area of mental health policy development and practice. Required cooperation between the sectors of education and social affairs has been developing in the country during the past decade. Medical interdisciplinary cooperation (pediatrics, family medicine, adult psychiatry) of CAP services is also continually increasing.

Appropriate views and attitudes in mental health services began to be adopted since 1990, when the Lithuania restored its independence. The recognition of the biopsychosocial paradigm necessitated the creation of new services and therapeutic approaches; this process remains an integral part of political, economic and social changes in society. For the last decades, Lithuania has had high suicide rates and poor mental health of children and adolescents. The first epidemiological study (18) has served as a reasonable basis for the proper developments in CAP research and service provision. The study revealed that 13.1% of children aged 7–16 years had mental health disorder; this was the first attempt in Baltic countries to get data and prevalence rates from the representative sample of the general school population (18).

Current CAP services are paid from the Compulsory Health Insurance Fund budget: primary outpatient personal health care services, consultations of medical specialists, CAP day care, and inpatient active treatment services. The main strengths and weaknesses are described in Table 1.

**Table 1:** The main strength and weaknesses in CAP service provision.

**Type of CAP service**	**Strengths**	**Weaknesses**
Inpatient active treatment and care	Sufficient quantity (5 units). Even distribution throughout the country.	Lack of continuing long term care after the discharge.Relapses and re-hospitalizations in complex cases due to insufficient duration and nature of care.
Day care	Usefulness was finally noticed and acknowledged by the authorities. Process of establishment of this type of services has begun.	Lack of funding and political will.At the beginning started to work in hospitals, therefore insufficiently developed in out patient clinics.
Mental Health Centers	Possibilities for multidisciplinary team work and collaboration with adult psychiatrists and family doctors. Continuity of professional assistance during transitional age from Childhood to adulthood.	Lack of specialized programs.Unequal access and availability throughout the country.
Private practice	Good possibility for clients and specialists. Serves to diminish stigma in society.	Unequal distribution, lack in rural areas.Expensive, lack of insurance payment.

The issues surrounding children's mental health are complex, and they include not only health but also education and the social environment. There is a lack of stable, mixed long-term funding for mental health prevention and intervention programs. The need for mixed funding models using insurance, state, and municipal budget target funds, and international project funding to develop and implement effective, modern CAP services for children and adolescents is officially declared but still not sufficiently implemented in Lithuania.

Health Insurance Fund tends to focus more on quantity than on the quality and effectiveness of CAP services. This shift is still insufficient in the organization and administration of daily clinical work. Specialized clinical programs in day care CAP services according to the age and specificity of the problems or disorders are in the country. Lithuania does not have any specialized CAP units for 0–3 and 3–7-year-old children. There is a lack of specialized programs for delinquent adolescents and long-term residential programs for those with complex psychosocial and developmental problems, aggressiveness, alcohol/drug abusers. Infant psychiatry is not developed as a separate field and offers no specialized services at all.

The primary mental health (psychiatric) care for children/adolescents and adults is provided in mental health centres. One hundred eleven mental health centres are operating in Lithuania, and their activities are organized in accordance with the guidelines and requirements set by the Ministry of Health. Mental health centres employ interdisciplinary teams of specialists: adult psychiatrists, child and adolescent psychiatrists, psychologists, social workers, and nurses. Some teams also have art, music, dance-movement, drama therapists successfully incorporated in the teamwork. These teams could serve as an excellent foundation for reaching adults and children/adolescents in the same service to tackle the transgenerational effects of parental mental illnesses on the psychosocial adjustment of the children of these parents.

### Initiatives and Programs for Children and Adolescents of Parents With Mental Health Disorders

Since 1990, no planned and systematic activities, programs, or interventions for COPMI were implemented into the clinical practice of CAP during the years of the country's independence. The relevance of this issue has been presented and discussed during national conferences of pediatricians, family doctors, child and adolescent psychiatrists, and adult psychiatrists. Some short-term initiatives arose in mental health services or municipalities, but without funding and administrational support, specific programs and systematic intersectoral multidisciplinary cooperation still have not been developed in everyday clinical practice. Children and adolescents lack age-appropriate information about the disorder of their parents and lack the possibility to address their worries and questions to an experienced specialist. In adult psychiatric services, attention to the clients' children is not routinely addressed. Good practices from other countries could be beneficial and serve as relevant models for the implementation and organization of help for COPMI.

Case managers in municipalities could be the primary persons organizing cross-sectoral cooperation according to the needs of each situation. Rarely and not systematically, mental health specialists in mental health centres invite children and adolescents of parents with a chronic mental illness to individual consultations or group activities. The implementation of day care programs in mental health centres could be an excellent possibility to organize specialized programs for these children and adolescents. The location of mental health centres in general outpatient clinics (polyclinics) helps diminish isolation as well as the stigma and perception of lacking social support. Usually pediatric and family medicine services are located in the same building and there are all possibilities for building long-term and systematic cooperation with somatic doctors. Sharing good examples would encourage the development of these initiatives in all mental health centres. Non-governmental organizations and the Lithuanian CAP Society could be an active part of these initiatives and developments. Decreasing stigma and raising awareness of society about sensitive COPMI topics and the complex needs of these families is also of utmost importance and still underdeveloped.

### Recommendations for CAP Services for the Further Developments in Organizing Help for COPMI

Special programs for COPMI should ensure a seamless integration of CAP services into the pediatric and family services and find ways for a systematic collaboration with adult psychiatry services. Active implementation of existing policy regulations and service quality assurance should remain the main directions for further development of the services. Funding for complex service provision and specialist training (team building and competencies) could be increased using the possibilities of more flexible financing in municipalities and government. Development of special treatment programs for COMPI (especially day care programs in mental health centres) according to the age and specificity of disorders or psychosocial actualities are essential. Family consulting and family therapy should be broader expanded and incorporated into CAP services. Active collaboration across non-governmental and governmental organizations should be more stimulated. Suggestions for CAP services in helping COPMI are presented in Table 2.

**Table 2:** Recommendations.

**Type of CAP service**	**Recommendations**
Inpatient active treatment and care	Routine asking about parents health. Direct to family-focused programs. Evaluations of psychosocial help are needed. Cooperation with adult psychiatrists and MHCs. Focus on building competencies of the team members (especially nurses, social workers, psychologists); practical skills in working with families.
Day care	Organize specialized programs for children and adolescents when their parents (caretakers) have mental health disorders. Include art therapies in day care programs. Share and implement good practices and initiatives.
Mental Health Centers	Find and screen children when their parents (caretakers) have mental health disorders. Organize therapeutic group activities for adolescents. Evaluations of psychosocial help are needed. Implementation of psychoeducational programs for parents about parenting and mental illness. Cooperation with municipalities and media. Usage of mobile mood-monitoring applications and remote electronic mental health care possibilities.
Private practice	Create mixed funding models and cooperate with CAP services, sectors of education, social affairs and NGOs. Participate in prevention, screening and early treatment initiatives with multiple public sectors.

Long-term programs reducing stigma, focusing on mental health promotion and prevention, should also be widespread in the country. Cooperation between the Ministry of Health and Education and Social Welfare systems should be elaborated more constructively and flexibly (comprising databases and funding). Active involvement of journalists and relevant opinion formation about mental disorders and COMPI could be helpful and serve more constructively in raising better understanding in society. Focus on the process of destigmatization of CAP involve media, public opinion, politics, specialists and CAP services. CAP services are a more favorable and less stigmatized area in Lithuania than adult psychiatry, so assistance and specialized programs for COPMI could be organized using the country's relatively well-developed CAP services network.

## Discussion

There are three main treatment approaches for COMPI listed in the JM REY'S IACAPAP e-Textbook: (1) Treatment of the ill parent; (2) Family Talk Intervention (comprising assessment of all family members, psychoeducation about the parent's disorder, connecting family history to psychoeducation, reduction of guilt and shame in children, enhancement of children's support) and (3) Support groups (1). Potijk et al. (19) note that “current interventions are aimed at the parents (psychoeducation and parenting skills), the children (psychoeducation, coping skills, mutual support), or the family as a whole (group sessions, online support)” and the “primary challenge is to increase the reach of the preventive interventions for parents and children.” In Lithuania, these interventions are sporadic and not systematic.

Maybery and Reupert (20) reported that “a large minority of adult psychiatric service patients are parents (from 12.2 to 45.0%).” Foster et al. (21) reviewing the support needs of families in psychiatric hospitals emphasize possibilities for extended and complex interventions, not only recovery-oriented care of the acutely ill patients: “recovery-oriented care can include the provision of family-focused care that supports the recovery of the parent-consumer and their family members and contributes to the prevention of intergenerational mental illness.” In Lithuania, family-focused care in adult psychiatric hospitals remains insufficient and not systematically organized. Research shows that not only psychiatrists but also other specialists in multidisciplinary mental health teams could play an active role in helping COMPI. In a study by Grant et al. (22), the authors described active participation of nurses stating that that “the capacity of nurses to support families has training, organizational and policy implications within adult mental health services in Ireland and elsewhere.”

Sharing international experiences of structural and organizational embedment of continued interventions for COPMI is essential. Even proper legislation takes time and administrative efforts to implement initiatives in daily practice. Norwegian experience (16) shows that “three years after the legislation was changed to making it mandatory to assess whether or not patients have children, it was still not fully incorporated in the routines of the entire workforce.” Another study made in the Netherlands (19) underlines that “in investigating how a preventive family-focused approach can be embedded in routine adult psychiatric care, a major barrier appeared to be the parents' hesitancy to let children participate in preventive programs.” Not only parents but also professionals have doubts and are sensitive to stigma issues that appear to serve as serious barriers to constructive implementation of prevention initiatives and help seeking (23–26). The topic of mental health and disorders is very sensitive and delicate in families and the society.

Some important issues were discussed by Wynter and Smith (27): “their study has explored the historical contexts to communicating mental health, and have stressed that money has always talked, for without adequate investment, services and care have suffered, contributing to the stigma surrounding mental illness.” Nyblade et al. (28) have indicated that “future investments in health facility stigma reduction should prioritize the involvement of clients living with a stigmatized condition or behavior and health workers living with stigmatized conditions and should address both individual- and structural-level stigma.”

It is essential to ask and hear the opinion of parents and children, and adolescents about their needs. Promoting user participation is an internationally outlined fundamental principle for CAP service organization: adolescents and children have the right to be informed about their health problems and participate in decision-making about their treatment (29). For those COMPI with mental disorders who need systematic and complex help, the promotion of their active participation in service organization and provision should be encouraged. International epidemiological research has revealed that children's self-reports for the comprehensive screening and early detection of mental health problems could be very valuable (30). Children's opinions and voices evaluating mental health are essential and helpful, together with parents and teachers' observations.

An international epidemiological study (31) conducted in 2010 showed that in five of the six European Union (EU) countries that participated in the study (including Lithuania), 10–15% of children aged 6–11 years had at least one mental health or behavioral disorder. The results of an international epidemiological study that examined self-reported mental health in children aged 6–12 years showed that in Lithuania, 22.1% of children had mental health disorders (30). The data of these studies revealed a high percentage of children's population as having mental disorders and a reasonable need for active CAP service development in Lithuania.

Helping COMPI in CAP services-specific screening and early detection interventions could be developed and implemented. Research data demonstrates a number of useful insights and connections found (32, 33). Current research findings should be effectively implemented in clinical practice and programs for COMPI.

The network of mental health centres in Lithuania provides services for both adults and children/adolescents. Minor organizational efforts and additional financial support are needed to systematically implement specialized programs for COMPI. There is a lack of planned interventions for the families in Lithuania and no research data or statistics monitoring who receives services for COMPI. Mental health services in Lithuania have many stigma-related factors that are needed to design and implement interventions both at the individual (patient, staff) and structural level (in health policy and the environment).

Another important theme is building mutual cooperation among adult psychiatric and CAP services. Difficulties in organizing CAP services are observed and described in various European countries (34); furthermore, there is a lack of specialists in this field in many parts of the world (35). A comprehensive review by Milestone Consortium et al. (36) examines the specificities of CAP and adult psychiatry in Europe and highlights the need for countries to organize a smooth transition for patients from children/adolescents to adult mental health services. Services for eating disorders should also be included in planning and organizing programs for COMPI. Research data (37) show that “eating disorders in parents predict eating disorders in children.” Pediatric and family medicine care could also serve as an appropriate opportunity for initiatives and integrative programs in finding ways to meet complex COMPI needs and assure their healthy development.

## Conclusion

The issue of finding ways to reach and help COMPI in mental health services is an actual and essential one. There is still a lack of appropriate long term systematic programs and ways to overcome complex organizational challenges. Sharing international experience and research in this field could help find principles and ways that best fit the situations in each country. In Lithuania, the potential of the existing network of CAP services and especially primary mental health centres could serve as a reasonable foundation for further developments in this field.

## Author Contributions

SL: preparation and writing of the manuscript.

## Conflict of Interest

The author declares that the research was conducted in the absence of any commercial or financial relationships that could be construed as a potential conflict of interest.

## Publisher's Note

All claims expressed in this article are solely those of the authors and do not necessarily represent those of their affiliated organizations, or those of the publisher, the editors and the reviewers. Any product that may be evaluated in this article, or claim that may be made by its manufacturer, is not guaranteed or endorsed by the publisher.
